# *In vitro* investigations on the impact of fermented dairy constituents on fecal microbiota composition and fermentation activity

**DOI:** 10.1128/spectrum.02193-24

**Published:** 2025-02-04

**Authors:** Qing Li, Angeliki Marietou, Freja Foget Andersen, Jiri Hosek, Carsten Scavenius, Jianbo Zhang, Clarissa Schwab

**Affiliations:** 1 Department of Biological and Chemical Engineering, Aarhus University, Aarhus, Denmark; 2 Department of Molecular Biology and Genetics, Aarhus University, Aarhus, Denmark; 3 Swammerdam Institute for Life Sciences, University of Amsterdam, Amsterdam, the Netherlands; University of Nebraska-Lincoln, Lincoln, Nebraska, USA

**Keywords:** fermented food, gut microbiota, fermentation, dairy, lactose, starter culture, butyrate

## Abstract

Fermented dairy constitutes a major dietary source and contains lactose as the main carbohydrate and living starter cultures, which can encounter the intestinal microbiota after ingestion. To investigate whether dairy-related nutritional and microbial modulation impacted intestinal microbiota composition and activity, we employed static fecal microbiota fermentations and a dairy model system consisting of lactose and *Streptococcus thermophilus* wild type and β-galactosidase deletion mutant. In addition, we conducted single-culture validation studies. 16S rRNA gene-based microbial community analysis showed that lactose increased the abundance of *Bifidobacteriaceae* and *Anaerobutyricum* and *Faecalibacterium* spp. The supplied lactose was hydrolyzed within 24 h of fermentation and led to higher expression of community-indigenous β-galactosidases. Targeted protein analysis confirmed that bifidobacteria contributed most β-galactosidases together with other taxa, including *Escherichia coli* and *Anaerobutyricum hallii*. Lactose addition led to higher (*P* < 0.05) levels of butyrate compared to controls, likely due to lactate-based cross-feeding and direct lactose metabolism by butyrate-producing *Anaerobutyricum* and *Faecalibacterium* spp. Representatives of both genera used lactose to produce butyrate in single cultures. When supplemented at around 5.5 log cells mL^−1^, *S. thermophilus* or its β-galactosidase-negative mutant outnumbered the indigenous *Streptococcaceae* population at the beginning of fermentation but had no impact on lactose utilization and final short-chain fatty acid profiles.

**IMPORTANCE:**

The consumption of fermented food has been linked to positive health outcomes, possibly due to interactions of food components with the intestinal microbiota. This study brings forward new insights into how major constituents of fermented dairy affect intestinal microbial ecology and activity when supplied together or alone. We provide evidence that lactose availability increased the production of butyrate by fecal microbiota through cross-feeding and did not observe a contribution of starter cultures to lactose metabolism, possibly due to a lack of competitiveness. The methodological setup used in this study can be implemented in future investigations to determine the impact of other fermented foods and their major components on intestinal microbiota composition and activity.

## INTRODUCTION

It has been estimated that 5%–40% of the human diet is fermented. In Europe and countries that mainly consume Western diets, fermented foods represent 17% of the diet (1). Diets rich in fermented foods, such as the Mediterranean diet, have been suggested to support longevity, health, and quality of life (2). The contribution of fermented food to the overall health benefits has been linked to raw ingredient modification and the formation of bioactive compounds during fermentation, as well as to interactions of fermented food with the immune system and the intestinal microbiota (2). Fermented foods contain live microbes, which might also affect the intestinal microbiome, yet defined interrelations between compositional and/or microbial components of specific fermented foods and intestinal microbiota response remain to be established (2).

Dairy products, including fermented dairy, account for approximately 14% of the energy intake of adults in Europe and North America (3). The major fermentable carbohydrate in dairy is lactose, and levels vary between unfermented and fermented products (4). Digestion of lactose by brush‐border lactase phlorizin hydrolases, as well as absorption, takes place in the small intestine. The ability of the host to break down lactose varies. In Northern Europe, up to 90% of the adult population are lactase persisters, while about 70% of the population worldwide are lactase non-persistent (5). Consumption of dairy products can lead to symptoms of intolerance in lactase non-persisters, e.g., bloating and diarrhea. It has been suggested that intestinal microbiota composition relates to the occurrence of intolerance due to distinctive fermentation activity, yet there is a possibility for microbiota adaptation to allow for the consumption of higher amounts of lactose (6, 7).

Undigested lactose can reach the lower gastrointestinal tract and be hydrolyzed by microbes that harbor the enzyme β-galactosidase (EC 3.2.1.23, glycosyl hydrolase family 2, GH2) to glucose and galactose, which then become available for intestinal microbial fermentation. From hexoses, fermenting microbes produce propionate or butyrate, or the fermentation intermediates, lactate, acetate, succinate, and formate, which can be further metabolized to propionate and butyrate through microbial cross-feeding (8). Intestinally produced short-chain fatty acids (SCFAs) and lactate are important mediators in the interactions between the microbiota and the host, contributing to intestinal homeostasis (9). Whether ingested lactose has an impact on intestinal microbiota fermentation activity has been investigated in a few *in vitro* and *in vivo* studies (10).

In addition to lactose, fermented dairy products often contain live starter cultures that frequently possess β-galactosidase activity. The β-galactosidase activity of starter cultures reduces the lactose content of the product (e.g., yogurt or fresh cheeses) and can potentially enhance the intestinal availability of lactose-derived monosaccharides *in vivo*. From a microbial ecology perspective and following the definition of Kinnunen et al. (11), “that any species not currently part of the resident community can be considered as a potential invader,” starter cultures that are ingested alive are possible invasive species to the intestinal microbiota (“resident microbiota”). Invasive species may cause changes to the abundance or composition of resident populations and might alter community functional properties (11). In the case of fermented dairy, starter cultures are ingested together with a major substrate, lactose. Whether co-application leads to a competitive advantage and/or affects the resident community remains to be established.

**Fig 1:**
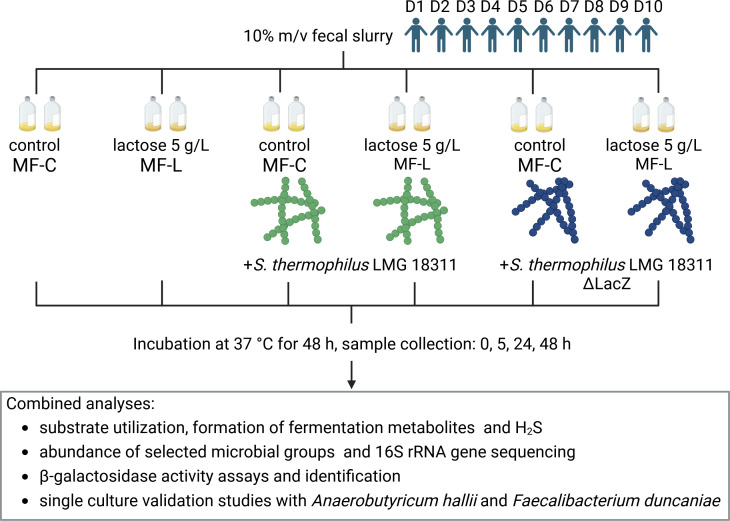
Experimental outline of fecal batch fermentations. Fecal microbiota of donors D1–D10 were fermented in Macfarlane medium with (MF-L) or without (MF-C) supplied lactose. Additionally, the β-galactosidase-positive *S. thermophilus* LMG 18311 (**D1–D10**) or negative *S. thermophilus* LMG 18311ΔLacZ (**D1–D6**) were added. Figure was prepared using Biorender.

In this study, we aimed to gain insight into the response of complex intestinal microbiota upon exposure to a model dairy system consisting of lactose and a starter culture with β-galactosidase activity. Employing a fermentation medium mimicking the complexity of nutrients that reach the proximal part of the colon together with fresh fecal microbiota as inoculum, we systematically determined the impact of lactose supplementation on community composition, β-galactosidase activity, metabolite cross-feeding, and fermentation profiles (Fig. 1). Additionally, we modulated the potential for lactose hydrolyzation by the addition of the β-galactosidase-positive dairy starter culture *Streptococcus thermophilus* and, for comparison, a β-galactosidase-negative mutant that lost the ability to hydrolyze lactose (Fig. 1).

## MATERIALS AND METHODS

### Donor recruitment and fecal sample processing

Fresh fecal samples were collected from 10 healthy donors (D1–D10) over a period of 5 months in Aarhus, Denmark in 2023. Anonymous sample collection and further processing were exempt from ethical approval according to the National Scientific Committee (National Videnskabsetisk Komité, Denmark). The donors were between 20 and 49 years of age, with regular eating patterns and bowel movements. Donors did not take any food supplements containing prebiotics or probiotics, nor did they use any medication affecting gut transit and digestion during the last 3 months preceding the sample donation. Donors provided written consent and collected samples independently following instructions. Each fecal sample was immediately transferred to a sealed bag containing an anaerobic GasPak (BD, Fisher Scientific, Roskilde, Denmark) and was processed within 4 h of defecation.

### Bacterial strains, cultivation, media, and growth kinetics

*Streptococcus thermophilus* LMG 18311 and its β-galactosidase-inactive mutant *S. thermophilus* LMG 18311ΔLacZ (12) were donated by Daniel Straume, Norwegian University of Life Sciences, Ås, Norway. *S. thermophilus* LMG 18311 was originally isolated from a commercial yogurt. *S. thermophilus* was routinely cultured on Todd-Hewitt glucose (THG) agar containing 3.7% (wt/vol) Todd-Hewitt powder, 0.8% (wt/vol) glucose, and 1.5% (wt/vol) agar or THG broth anaerobically at 42°C. All media ingredients were purchased from Merck (Søborg, Denmark) unless otherwise stated.

To determine the impact of lactose on the growth of *S. thermophilus* strains, cultures were grown in Hogg-Jago broth containing 3% tryptone, 1% yeast extract, 0.2% meat extract, 0.5% KH_2_PO_4_, and either no supplemented carbohydrate (HJ), 0.5% glucose (approximately 23 mM, HJG), 0.5% lactose (12 mM, HJL), 0.5% galactose (20 mM, HJGa), or 0.5% glucose and lactose (22 and 11 mM, respectively, HJGL). Growth was monitored using a Grant-bioDEN-1 McFarland densitometer (Grant Instruments, Royston, England). Samples (1 mL) were collected at 0, 4, 9, and 24 h and centrifuged (10 min, 10,000 × *g*). The cell pellets and supernatants were stored separately at −20°C for further analysis.

To prepare cultures for the inoculation of fecal batch fermentations, *S. thermophilus* strains were grown anaerobically in HJGL for 24 h at 37°C, centrifuged, and resuspended in 50 mM phosphate-buffered saline (PBS), pH 6.5. To determine colony-forming units (CFU), liquid cultures were serially diluted in PBS and plated on THG agar plates.

*Anaerobutyricum hallii* DSM 3353 and *Faecalibacterium duncanie* DSM 17677 were obtained from the German Collection of Microorganisms and Cell Cultures (DSMZ, Braunschweig, Germany) and were grown in modified yeast casitone fatty acid medium supplemented with 50 mM glucose (YCFA-G) (13). To determine the growth and β-galactosidase activity of *Anaerobutyricum* and *Faecalibacterium* strains in the presence of lactose, the strains were grown in YCFA supplemented with 50 mM lactose (YCFA-L) and compared to growth in YCFA-G (only *A. hallii* DSM 3353). Turbidity was monitored using a McFarland densitometer. Samples (1 mL) were collected at 0 and 24 h and centrifuged (10 min, 10,000 × *g*). Cell pellets and supernatants were stored separately at −20°C for further analysis.

### Static fecal microbiota batch fermentations

Batch fermentations were conducted in a modified medium (14) that was based on the medium of Macfarlane et al. (15). The modified Macfarlane medium contained complex carbohydrates and nitrogen sources to mimic substrates that are available in the large intestine and was prepared with additional buffer capacity for *in vitro* fermentations. The control medium (MF-C) contained (in g L^−1^) 1.0 cellobiose, 1.0 xylan, 1.0 arabinogalactan, 0.5 inulin, 1.0 soluble starch, 3.0 amicase, 5.0 bacto tryptone, 1.5 meat extract, 4.5 yeast extract, 4.0 porcine mucin, 0.005 hemin, 0.4 bile salts, 3.0 KH_2_PO_4_, 9.0 NaHCO_3_, 0.6 MgSO_4_, 0.1 CaCl_2_·2H_2_O, 0.2 MnCl_2_·4H_2_O, 0.005 FeSO_4_·7H_2_O, 0.1 ZnSO_4_·7H_2_O, 4.5 NaCl, and 4.5 KCl. Tween 80 (1 mL) was added together with a 10-fold-diluted MEM Vitamin Solution (100×). Additional SCFA was supplied at a final concentration of 33 mM acetate, 1 mM isobutyrate, isovalerate, and valerate, and 9 mM propionate, as the presence of selected SCFA has been shown to support the growth of individual microbial gut microbes *in vitro* (16). MF-L contained approximately 5 g L^−1^ (or 10 mM) lactose as present in bovine milk. Before boiling, the pH was adjusted to pH 7.1. During cooling down, the medium was flushed with CO_2_, and cysteine-HCl was added (1 g L^−1^) before transferring 20 mL of medium to 50 mL serum flasks under CO_2_ atmosphere. Flasks were sealed with rubber stoppers and aluminum caps and autoclaved.

For fecal slurry preparation, 1 g of fresh fecal sample was resuspended in 10 mL of anaerobically prepared peptone water in an anaerobic bench to obtain a 10% (m/vol) solution. The fecal slurry solution was inoculated in MF-C and MF-L at 1% (vol/vol) inoculum level. To determine the impact of starter cultures on fermentations, *S. thermophilus* LMG18311 or LMG 18311Δ*LacZ* were added to donor fermentations D1–D10 and D1–D6, respectively. The mutant strain LMG 18311Δ*LacZ* was only added to fermentations D1–D6, as we observed no difference between the two strains in the first six fermentations. Fermentations were conducted for 48 h at 37°C. Samples (1 mL) were collected at times 0, 5, 24, and 48 h, centrifuged, and cell pellets and supernatants were stored at −20°C until further analysis. The majority of fermentations were run in independent duplicates, while some samples were run as single cultures.

### Substrate, metabolite, and hydrogen sulfide analysis

Substrate use and metabolite formation were determined with high- or ultra-performance liquid chromatography with a refractive index detector (HPLC-RI and UPLC-RI). Supernatants collected from pure cultures or fecal fermentations were diluted with 5 mM H_2_SO_4_ or MilliQ water and filtered with PTFE 0.45 µm pore size filters (Agilent Technologies, Glostrup, Denmark). Fermentation metabolites were separated using an Agilent 1260 LC system equipped with a Hi-Plex H column (300 × 7.7 mm, 8 µm particle size) with a guard column (both Agilent) with 5 mM H_2_SO_4_ as eluent and a flow rate of 0.6 mL min^−1^ at 40°C (17) (HPLC-RI). Lactose, glucose, and galactose levels were determined using a Thermo Fisher Vanguard system (UPLC-RI, Thermo Fisher Scientific) equipped with an Aminex HPX-42A column (Bio-Rad, Copenhagen, Denmark). MilliQ was used as an eluent at a flow rate of 0.6 mL min^−1^ at 40°C. External standards were used for the quantification of sugars and fermentation metabolites.

The production of H_2_S was determined photometrically for samples collected from donors D4–D10 at 48 h based on the reaction of H_2_S with N, N-dimethyl-1,4-phenylendiamine that produces methylene blue in the presence of iron (III) (Fe^3+^) as described previously (18). Samples were analyzed in technical triplicates.

### Determination of β-galactosidase activity and targeted proteomics

To determine β-galactosidase activity, pellets obtained from *S. thermophilus* cultures at 0, 4, 9, and 24 h of incubation in HJ, HJG, HJGa, HJL, and HJGL were resuspended in 500 µL of phosphate buffer (PB, 50 mM, pH 6.5) and lysed by bead beating (Fastprep-24, MP Biomedicals, Kaastrup, Denmark) for 40 s at 6 ms^−1^. Cell pellets obtained from *Anaerobutyricum* and *Faecalibacterium* cultures after 24 h of incubation in YCFA-G and YCFA-L, as well as fecal batch fermentations at 48 h, were processed in the same way. The lysates were centrifuged at 14,000 × *g* for 5 min, and the supernatants were stored at −20°C until further analysis.

The β-galactosidase activity was determined spectrophotometrically by adding 10 µL of cell extract to 90 µL of 2 mM oNPG (2-nitrophenyl-β-D-galactopyranoside) in a 96-well microplate and measuring the absorbance at 405 nm over a period of 30 min with 1 min intervals and 10 s shaking before every measurement. A nitrophenol standard curve was included with a range of 0.02–2.5 mM.

To visualize active β-galactosidases, the proteins of the cell extracts were separated using SDS-PAGE (4%–12% gradient; SurePAGE, Genescript, Oxford, UK). Cell extracts (45 µL) were mixed with 15 µL of loading dye (4× LDS sample buffer, Genescript) with no heating. Two gels were prepared for each sample; one was stained with Instant Blue Coomassie Protein Stain (Abcam, Fisher Scientific) following standard protocols, and the other was stained with methyl-umbelliferyl-β-D-galactose (MUG) as described (19). Molecular weight was implied using Broad Multi Color Pre-Stained Protein Standard (Genescript).

Bands with β-galactosidase activity were excised and stored at −80°C until preparation for LC-MS/MS with in-gel digestion (20). Tryptic peptides were micro-purified using Empore SPE C18 disks packed in 10 µL pipette tips (21). LC-MS/MS analyses were performed using an Easy nLC 1200 connected to a Tribrid Eclipse mass spectrometer (both Thermo Fisher Scientific, Waltham, MA, USA). The samples were suspended in 0.1% formic acid, injected, trapped, and desalted on a precolumn (ReproSil-Pur C18-AQ 1.9 µm resin, Dr. Maisch GmbH, Germany). The peptides were eluted and separated on a 20 cm analytical column (75 µm i.d.) packed with ReproSil-Pur C18-AQ 1.9 µm resin in a pulled emitter. Peptides were eluted at a flow rate of 250 nL min^−1^ using a 20 min gradient from 5% to 40% of solution B (0.1% formic acid and 90% acetonitrile). The collected MS files were converted to mascot generic format (.mgf) using Mascot Distiller (Matrix Science). The generated peak lists were searched against the uniport database with taxonomy restriction (TAX ID: 1681, 1680, 216816, 1685, 562, 1304, 1308, 39488, 40520, 1352, 1351, 1502, 853, 411483, 301301, 166486, 116085, 66219, 47678, 818, 817, 52227, and 214856) using an in-house Mascot 2.8.2 search engine (matrix science). Search parameters allowed one missed trypsin cleavage site with peptide tolerance and MS/MS tolerance set to 10 ppm and 50 mmu, respectively, and β-galactosidase-related peptide profiles were manually checked for specificity.

### DNA extraction, quantitative PCR, and 16S rRNA gene sequencing

Using the FastDNA SPIN Kit for Soil (MP Biomedicals), DNA was isolated from cell pellets after 0 and 48 h of batch fermentation. From *t* = 0 h, DNA was isolated from one of the duplicates, while at 48 h, DNA was isolated and analyzed from both samples. No biomass for DNA collection was obtained from fermentations of D3. Cells were lysed by bead beating for 40 s at 6 ms^−1^, and DNA was eluted in 50 µL TE buffer and diluted 10-fold before use. Quantitative PCR (qPCR) was used to determine the total bacterial cells and the abundance of *Streptococcaceae* using primer pairs 338F (5′-ACTCCTACGGGAGGCAGCAG-3′) and 534R (5′-ATTACCGCGGCTGCTGG-3′), and STF (5′-ACGCTGAAGAGAGGAGCTTG-3′) and STR (5′- GCAATTGCCCCTTTCAAATC-3′) as described (17).

For the 16S rRNA gene library preparation, we used a two-step PCR approach according to the Illumina 16S Metagenomic Sequencing Library Preparation guide (Illumina, San Diego, USA) as described (18). Briefly, the 16S rRNA gene was amplified using Bac341F and Bac805R with adapters. A second PCR was used for barcoding the samples. Sequencing was performed on a MiSeq sequencer (Illumina) at the Section of Microbiology at Aarhus University. All samples were analyzed in the same run and included a negative control (mock DNA isolation procedure). From *t* = 0 h, only one sample per treatment was sequenced.

### Processing of 16S rRNA gene libraries

Primer sequences were removed using cut adapt (version 4.4; -O 12 --discard-untrimmed -g TCGTCGGCAGCGTCAGATGTGTATAAGAGACAGCCTACGGGNGGCWGCAG -G GTCTCGTGGGCTCGGAGATGTGTATAAGAGACAGGACTACHVGGGTATCTAATCC --pair-adapters --minimum-length 75) (22), and only inserts that contained both primers and were at least 75 bases were kept for downstream analysis. Reads were quality filtered using the filterAndTrim function of the dada2 package (maxEE = 2, truncQ = 3, minLen = 150, trimRight = 40, trimLeft = 40). The learnErrors and dada functions were used to calculate sample inference using pool = pseudo as a parameter. Reads were merged using the mergePairs function and bimeras were removed with removeBimeraDenovo (method = pooled). The remaining amplicon sequence variants were taxonomically annotated using the IDTAXA classifier (23) in combination with the Silva version 138 database (24). The median number of reads per processed sample was 27,056 (range 4,272–44,967 reads), and the negative control yielded 95 reads. Two samples were removed from further analysis as sequencing failed.

### Statistical analysis

The normality of the data was assessed using the Shapiro-Wilk test. The non-parametric methods, Kruskal-Wallis *H* test with Mann-Whitney pairwise testing, were used to test for differences in cell counts determined by qPCR. *t*-test was used to compare the α-diversity indices and pH values of fermentations at 48 h. All statistical tests were implemented in PAST (25).

Statistical analysis of 16S rRNA gene amplicon libraries was conducted with QIIME 2 version 2023.9.1 (26). Alpha‐diversity metrics (observed features and Chao and Shannon index, β-diversity metrics, unweighted UniFrac, and principle coordinate analysis) were estimated using q2‐diversity after samples were rarefied (subsampled without replacement) to 9,558 sequences per sample. The threshold was 10,467 reads in case of analysis of data at 48 h only. Beta-diversity significance was assessed with q2-diversity package based on the PERMANOVA method and 999 permeations. Differential expression analysis of the relative abundance data of microbial families and genera was performed in R using DESeq2 (27). DESeq2 employs Wald *t*-test and corrects for multiple testing with the Benjamini-Hochberg testing.

## RESULTS

### Lactose addition enhanced the abundance of *Bifidobacteriaceae, Anaerobutyricum*, and *Faecalibacterium*

**Fig 2:**
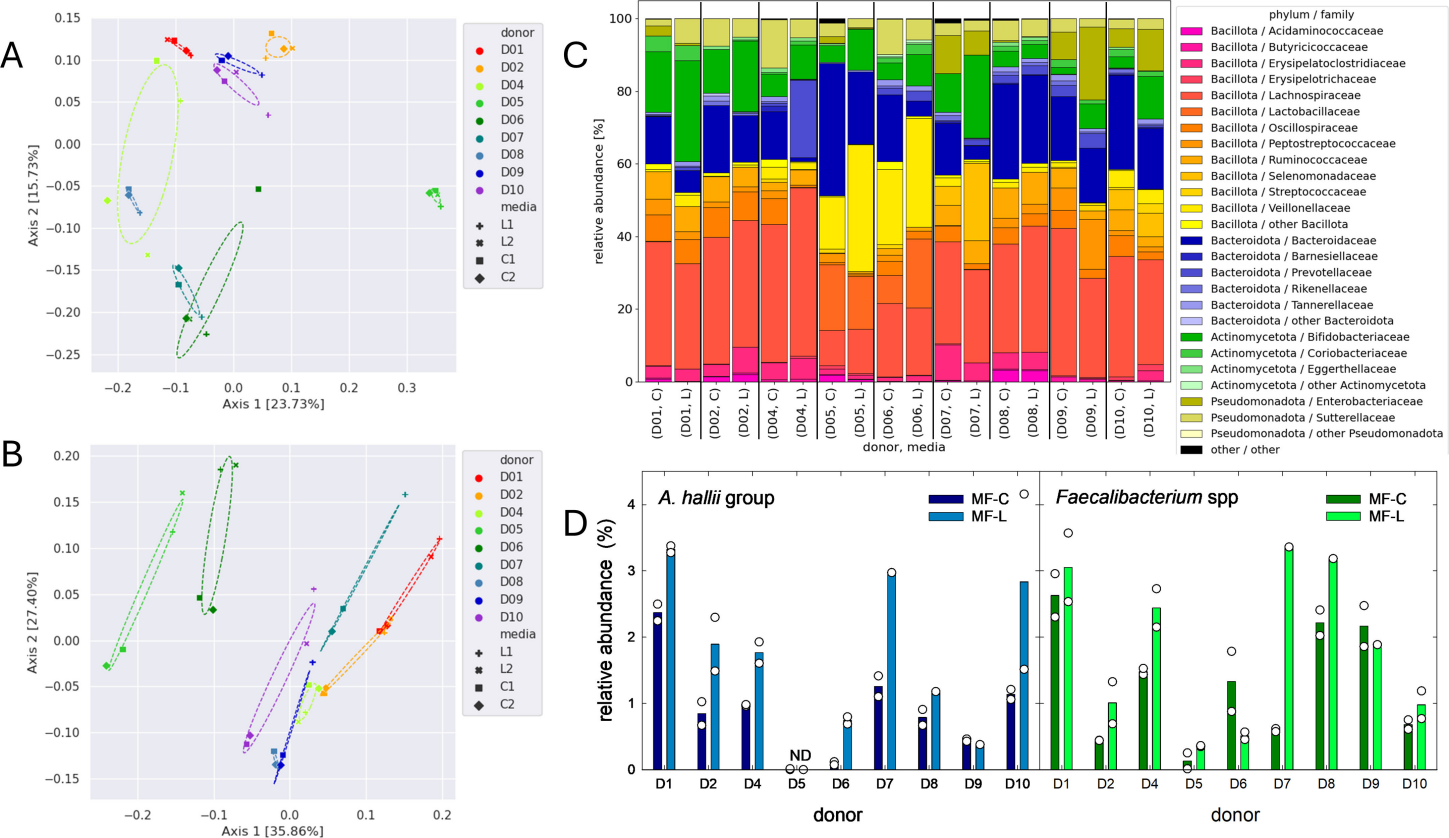
Impact of lactose supplementation on microbiota composition. Fecal slurries were incubated in MF-C or MF-L in duplicate fermentations, and community composition was determined using 16S rRNA gene amplicon sequencing after 48 h of incubation at 37°C. (**A and B**) Beta-diversity according to unweighted (**A**) and weighted (**B**) UniFrac analysis. For each donor, both fermentation replicates were included in the analysis. (**C**) Average relative abundance profiles of major bacterial families in MF-C (**C**) and MF-L (**L**). (**D**) Average relative abundance of the *Anaerobutyricum hallii* group (left plot) and *Faecalibacterium* spp. (right plot) in MF-C and MF-L; relative abundance of fermentation replicates is shown as white circles. ND, not detected.

We determined the impact of lactose addition on microbiota composition after 48 h fermentations in Macfarlane medium without and with supplied lactose. In total, the community composition of nine fecal donor microbiotas (D1, D2, and D4–D10) was analyzed. Based on β-diversity analysis, donor microbiota individuality was a strong determinant of microbiota composition based on unweighted (Fig. 2A) and weighted (Fig. 2B) UniFrac after 48 h of incubation (*P* = 0.001, PERMANOVA). The presence of lactose (MF-C versus MF-L) significantly affected composition based on weighted (*P* = 0.005) but not on unweighted UniFrac (*P* = 0.275). The Shannon index was higher in fermentations in MF-C than when lactose was present (MF-L) (5.7 ± 0.3 and 5.3 ± 0.5, *P* < 0.05, Mann-Whitney test, Table S1), while there were little differences in the Chao indices (232 ± 45 and 206 ± 49) (Table S2).

After 48 h of fermentation, there was a significant (*P*_adj_ = 0.028, Wald *t*-test implemented in Deseq2 with Benjamini-Hochberg multiple test correction) increase in the relative abundance of *Bifidobacteriaceae* (median increase + 6.7%, interquartile range [IQR] 25% and 75%: IQR25 + 3.7%, IQR75 + 8.5%] in MF-L compared to MF-C (Fig. 2C; Table S3). In contrast, the relative abundance of *Desulfovibrionaceae* was lower (*P* < 0.001, –0.01%, IQR25 −0.15%, IQR75 −0.0%). On a genus level, *Bilophila Eisenbergiella,* and *Parasutterella* were significantly less abundant (*P*_adj_ < 0.05) in fermentations grown in MC-L, and there was a trend for lower abundance of *Alistipes* and UCG-009 (*P*_adj_ <0.1). (Table S3). The relative abundance of *Veillonellaceae* was higher after incubations in MF-L in fermentations of donors D6 and D7, while the abundance of *Enterobacteriaceae* was higher for D9 and D10 compared to MF-C. On a genus level, the median abundance of *Anaerobutyricum* and *Faecalibacterium* spp. was higher in samples incubated in MF-L than MF-C (+0.8%, IQR25 + 0.2%, IQR75 + 1.1%, and +0.4, IQR25 + 0.0%, IQR75 + 1.0%, respectively) (Fig. 2D).

### Lactose addition enhanced the formation of butyrate by all donor microbiota and reduced levels of H_2_S

**Fig 3:**
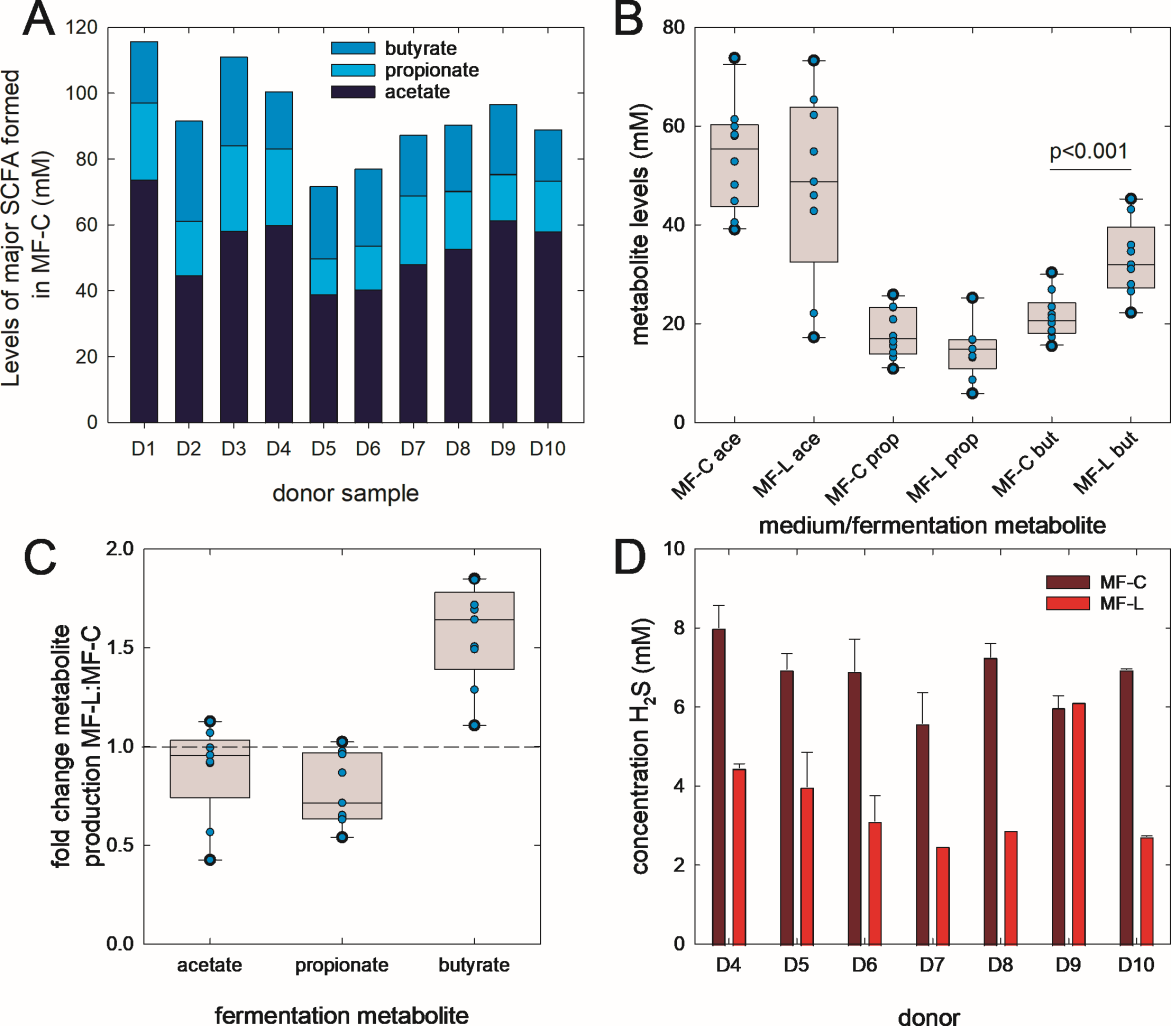
Formation of fermentation metabolites and H_2_S. Fecal slurry batch fermentations were performed using MF-C and MF-L at 37°C for 48 h. Levels of major SCFA were determined using HPLC-RI, while H_2_S formation was analyzed using a colorimetric assay. (**A**) Profiles of major SCFA acetate, propionate, and butyrate formed in MF-C. (**B**) Levels of acetate (ace), propionate (prop), and butyrate (but) after growth in MF-C and MF-L. Mann-Whitney test was used to identify statistical difference between SCFA formed with different treatments, *P* < 0.05 was considered significant. (**C**) Fold change in metabolite production (acetate, propionate, and butyrate) after incubation in MF-L compared to MF-C. (**D**) Levels of H_2_S produced by donor fermentations D4–D10; samples were analyzed in technical triplicates.

Next, we investigated the impact of lactose addition on the fermentation activity of fecal microbiota of donors D1–D10. The initial pH of the medium was 6.58–6.60. After fermentation, the pH of MF-C (6.25 ± 0.11) was significantly higher than MF-L (5.98 ± 0.20). In MF-C, the major SCFA was acetate (49%–64%), followed by butyrate (16%–33%) and propionate (15%–23%); total SCFA levels ranged from 72 to 116 mM (Fig. 3A). While there was no significant difference in acetate and propionate levels formed during incubation in MF-C and MF-L, there was significantly more butyrate produced in MF-L (*P* < 0.005, Mann-Whitney test) (Fig. 3B). In MF-L, butyrate levels increased in all samples (median 1.57-fold increase [IQR25 1.49, IQR75 1.72]) (Fig. 3C). The levels of H_2_S were determined in samples D4–D10, as we observed that the addition of a fermentable carbohydrate reduced H_2_S formation *in vitro* in a previous study (18). The release of H_2_S was significantly lower (*P* < 0.005, Mann-Whitney test) in samples incubated in MF-L compared to MF-C. Except for D9, there was less H_2_S detected when lactose was present (MF-L) compared to MF-C in the tested samples (Fig. 3D).

### The presence of lactose induced β-galactosidase activity

**Fig 4:**
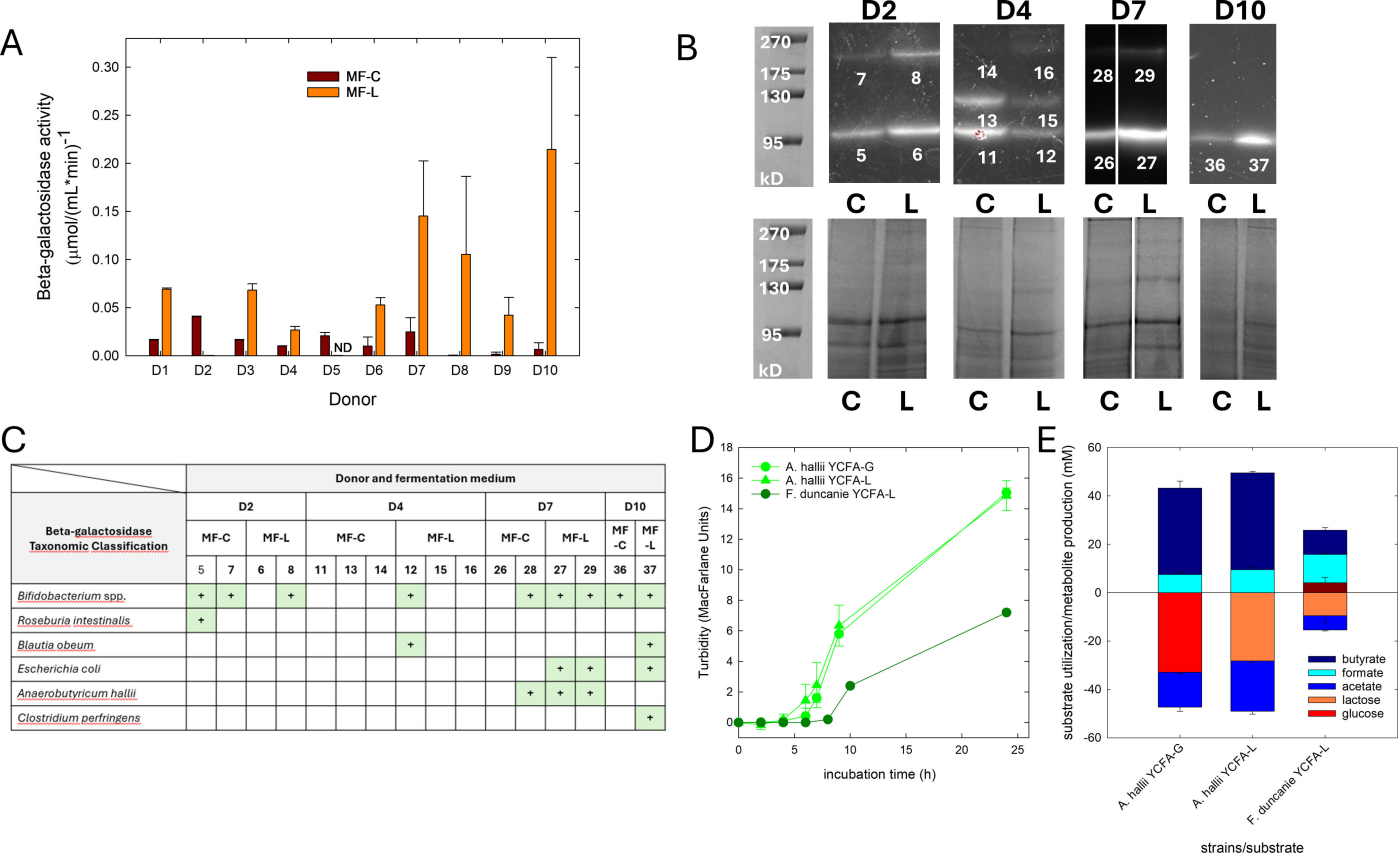
Fecal microbiota β-galactosidase activity and lactose utilization of selective gut microbes. Fecal slurry batch fermentations were performed using MF-C and MF-L at 37°C for 48 h. (**A**) Beta-galactosidase activity was quantified using oNPG, and (**B**) active β-galactosidases were visualized by SDS-PAGE followed by MUG staining of cell extracts of donors D2, D4, D7, and D10 (upper panel). Each band visualized with MUG was assigned a running number. For comparison, gels were also stained with Coomassie blue (lower panel); left plot, molecular weight standard in kDa. (**C**) Active bands were excised from the gels and β-galactosidase-related peptide fragments were identified by LC-MS/MS. (**D**) *A. hallii* DSM 3353 and *F. duncaniae* DSM 17677 were grown in YCFA-G and YCFA-L, and turbidity was monitored using a Mcfarland densiometer (left plot); substrate utilization and metabolite formation after 24 h were determined using UPLC-RI (lactose and glucose) and HPLC-RI (fermentation metabolites) (right plot) (E).

Gut microbes utilize lactose with the help of intra- or extracellular β-galactosidases or intracellular β-phospho-galactosidases. We determined the impact of lactose availability on the β-galactosidase activity of fecal fermentations using oNPG. oNPG activity ranged from 0 to 0.215 µmol mL^−1^ min^−1^. Compared to cell extracts derived from MF-C, 80% of the cell extracts from MF-L had 3- to 222-fold higher β-galactosidase activity when oNPG was used as substrates (Fig. 4A). In addition, we separated protein extracts on SDS-PAGE and stained gels with MUG to identify proteins with potential β-galactosidase activity. Higher fluorescent signals in most samples incubated with MUG suggested induction of β-galactosidase activity by the presence of lactose (MF-L) compared to MF-C in agreement with oNPG measurements (Fig. 4B; Fig. S1). The size of the active bands that were indicative of the presence of β-galactosidases differed between samples and treatments and were observed in a weight range of 95 to >175 kDa (Fig. 4B; Fig. S1). Bands showing β-galactosidase activity were excised from the gels derived from donors D2, D4, D7, and D10, and the protein composition was determined using LC-MS/MS. Among the active fractions (Fig. 4B), we identified peptides with homologies to *Bifidobacterium* spp. β-galactosidases for each donor in fermentations that were derived from MF-C (except D4) and MF-L. In samples from MF-L, we observed additional β-galactosidase-associated peptide fragments of *Blautia obeum* (D4 and D10)*, E. coli* (D7 and D10)*, A. hallii* (D7), and *Clostridium perfringens* (D10) (Fig. 4C).

### *Anaerobutyricum* spp. and *Faecalibacterium duncanie* use lactose to produce butyrate

We noticed that relative abundances of the *A. hallii* group and *Faecalibacterium* were higher in most samples fermented in MF-L compared to MF-C. Both genera contain members that are able to produce butyrate. In addition, the genomes of the type strains *Anaerobutyricum hallii* DSM 3353 and *Faecalibacterium duncanie* DSM 17677 harbor genes encoding glycoside hydrolase family 2 enzyme with predicted β-galactosidase activity (accession ID WP_005347370 and ATP00898.1 on NCBI). The β-galactosidases of *A. hallii* and *F. duncaniae* had a predicted size of 119 and 116 kDa, respectively. Phylogenetic analysis suggested a close relationship with enzymes of *Coprococcus catus* and *Blautia schinkii,* and *Blautia* spp. and *Roseburia hominis*, respectively (Fig. S2).

To determine whether *A. hallii* DSM 3353 and *F. duncaniae* DSM 17677 used lactose to produce butyrate, we grew strains in YCFA-G and YCFA-L. The growth of *A. hallii* was similar in YCFA-G and YCFA-L (Fig. 4D). *A. hallii* used 68% of the provided lactose together with acetate to produce butyrate and formate in a ratio of approximately 4.5:1; accumulation of galactose was not observed. *F. duncaniae* consumed 23% of the provided lactose, released some galactose, and produced butyrate and formate (Fig. 4D). After growth in YCFA-L, β-galactosidase activity of *A. hallii* (18.2 ± 0.7 nmol mL^−1^ min^−1^) was higher than in YCFA-G (0.8 ± 0.2 nmol mL^−1^ min^−1^) (*P* < 0.05). The β-galactosidase activity of *F. duncaniae* was 38.1 ± 1.4 nmol mL^−1^ min^−1^.

### Addition of the starter culture *S. thermophilus* impacted initial community composition and had little effect on fermentation activity

To evaluate whether the addition of β-galactosidase-positive *S. thermophilus* impacted community composition, lactose utilization, and overall fermentation activity, we supplied *S. thermophilus* LMG 18311 (D1–D10) and its β-galactosidase inactivation mutant LMG 18311Δ*LacZ* (D1–D6) to fecal batch fermentations. In single cultures, only *S. thermophilus* LMG 18311 grew in the presence of lactose (Fig. S3A and B) and expressed β-galactosidase (Fig. S3C and D). Beta-galactosidase activity was highest when *S. thermophilus* LMG 18311 was grown in HJL and HJGL, with low activity observed during growth in HJG (Fig. S3C). *S. thermophilus* LMG 18311 used lactose when available as a sole carbohydrate source or when supplied together with glucose to mainly produce lactate (Fig. S4).

**Fig 5:**
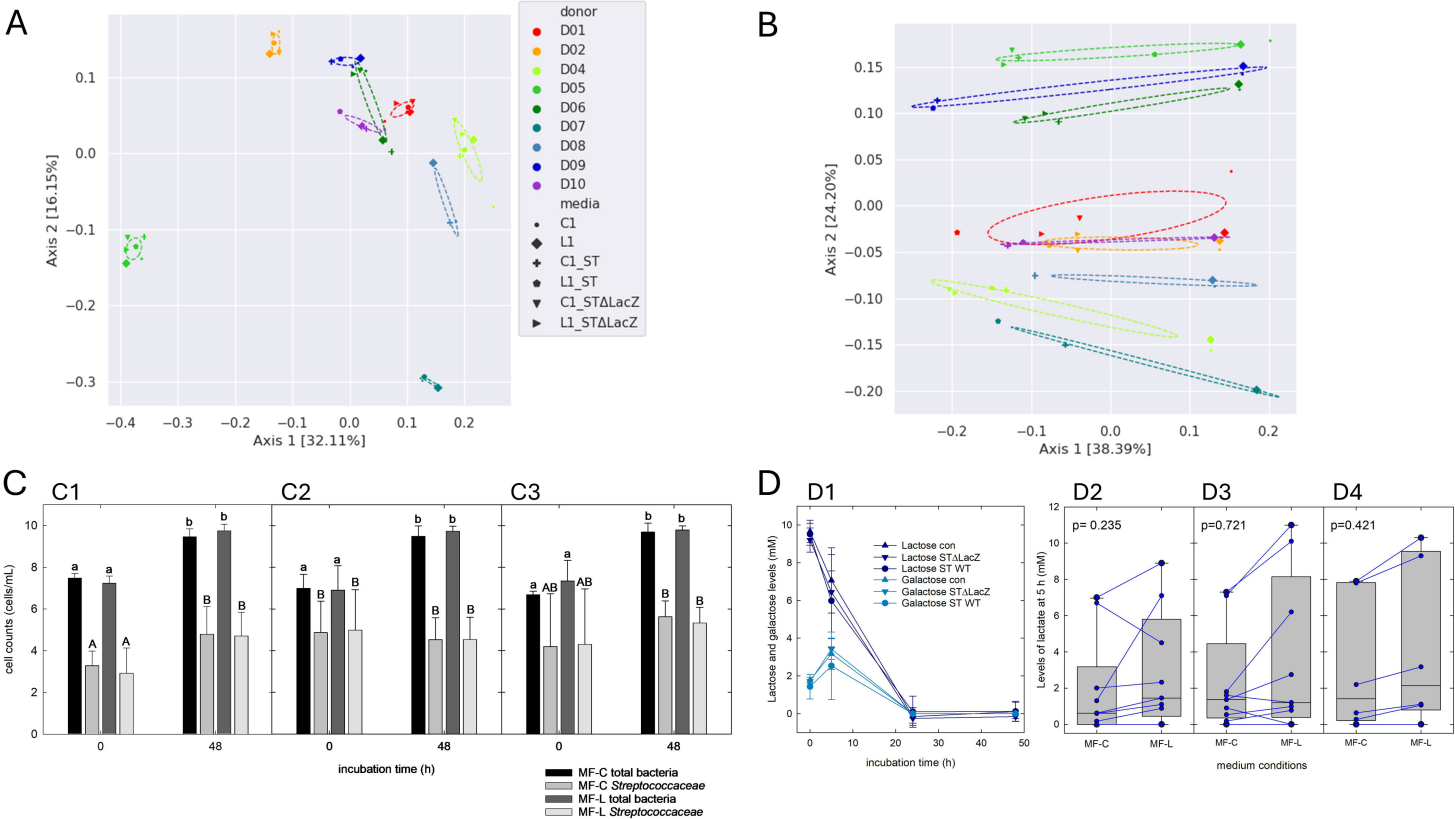
Impact of starter culture addition on microbial community composition and fermentation activity. Microbial community composition and abundance of *Streptococcaceae* were determined using 16S rRNA gene amplicon sequencing and qPCR, respectively. Levels of lactose, galactose, and lactate were determined in fecal batch fermentations incubated at 37°C for 48 h by UPLC-RI and HPLC-RI. (**A and B**) Unweighted (**A**) and weighted (**B**) UniFrac analysis of samples collected at *t* = 0 h with and without the addition of *S. thermophilus* LMG 18311 and LMG 18311∆*LacZ*. (**C**) Cell counts of total bacteria and *Streptococcaceae* determined by qPCR in control fermentations (**C1**), fermentations supplemented with *S. thermophilus* LMG18311 (**C2**), or *S. thermophilus* LMG1833 18311∆LacZ (**C3**). Different small and capital letters indicate significant differences in cell counts of total bacteria and *Streptococcaceae*, respectively, at *t* = 0 and/or *t* = 48 h. Differences in abundance were identified using Kruskal-Wallis *H* test followed by Mann-Whitney pairwise testing. A *P* < 0.05 was considered significant. (**D**) Lactose, galactose, and lactate levels during fermentation. Lactose and galactose levels during fermentations in MF-L without or with supplements of *S. thermophilus* LMG 18311 and LMG 18311∆LacZ. (**D2–D4**) Lactate levels during fermentation in MF-C and MF-L without culture addition (**D2**), and fermentations supplied with *S. thermophilus* LMG 18311 (**D3**) and *S. thermophilus* LMG 18311Δ*LacZ* (**D4**). Differences in lactate levels formed in MF-C and ML-L in control (**D2**) and fermentations with *S. thermophilus* LMG 18311 (**D3**) and *S. thermophilus* LMG 18311Δ*LacZ* (**D4**) were identified using Mann-Whitney test. A *P* < 0.05 was considered significant.

Initial microbiota composition was distinctive between donors according to unweighted (Fig. 5A) and weighted UniFrac (Fig. 5B) (*P* = 0.001, PERMANOVA). The addition of *S. thermophilus* significantly impacted the β-diversity of the communities at *t* = 0 h (Fig. 5C) as indicated by weighted UniFrac analysis (*P* = 0.001). Total bacteria counts at the beginning of batch fermentations were 7.0 ± 0.9 log cells mL^−1^. *S. thermophilus* LMG 18311 and LMG 18311Δ*LacZ* were added at 5.8 ± 0.7 and 5.4 ± 0.6 log CFU mL^−1^. Based on the results obtained with qPCR, average counts of *Streptococcaceae* in samples inoculated with LMG 18311 (4.9 ± 1.7 log cells mL^−1^) and 18311Δ*LacZ* (4.3 ± 2.4 log cells mL^−1^) were significantly higher than in the uninoculated controls (3.1 ± 1.0 log cells mL^−1^) at *t* = 0 h (Fig. 5C1 through C3). When *S. thermophilus* LMG18311 (Fig. 5C2) and 18311Δ*LacZ* (Fig. 5C3) were supplied, the *Streptococcaceae* constituted between a median of 30.6% (IQR25 + 25.9%, IQR75 + 31.9%) and 28.5% (IQR25 + 0.3, IQR75 +33.3) of the microbial population at the beginning of fermentation, respectively (Fig. S5). After 48 h of fermentation, total bacterial cell counts increased by about 2.5 log cells mL^−1^ (9.6 ± 0.4 log cells mL^−1^), while the abundance of *Streptococcaceae* remained low (4.7 ± 1.2 log cells mL^−1^) and was not different (Kruskal-Wallis test), regardless of the addition of *S. thermophilus* or lactose (Fig. 5C1 through C3). At 48 h, the pH of fermentations MF-C (mean pH 6.22–6.26) or MF-L (5.96–6.02) did not differ from fermentations that were not supplemented with *S. thermophilus*. As the addition of *S. thermophilus* might increase the rate of lactose metabolism, we recorded the concentrations of lactose and lactate at 5 and 24 h after the initiation of the fermentation. There was no difference in lactose utilization between treatments at 5 h, and there was a slight accumulation of galactose in all samples (Fig. 5D1). In both supplemented and unsupplemented fermentations, lactose and galactose were used within the first 24 h of fermentation (Fig. 5D1). While the presence of lactose (MF-L) led to higher lactate levels at 5 h compared to MF-C in most samples, there was no significant difference in lactate levels between samples incubated in MF-C or MF-L, regardless of the supplementation with *S. thermophilus* LMG 18311 or LMG 18311∆LacZ when tested using Kruskal-Wallis (*P* = 0.488) or Mann-Whitney pairwise test (Fig. 5D2 through D4). At 24 h, lactate was detected only in fermentations of D1 and D2 (1.4 mM).

## DISCUSSION

### *Bifidobacteriaceae* are immediate responders to lactose availability

The disaccharide lactose is accessible to fermentation after hydrolysis. Strains of *Bifidobacterium longum* subsp. *longum*, *Bifidobacterium adolescentis*, *Bifidobacterium pseudocatenulatum,* and *Bifidobacterium bifidum* are prevalent species in the adult gut and can readily ferment lactose to mainly acetate, lactate, and formate. Previous studies suggests that some strains even prefer lactose over glucose (28). *In vivo*, there was an increase in fecal *Bifidobacterium* abundance in the majority of obese individuals or in lactase non-persisters upon consumption of lactose (6, 29, 30). In another study that included lactase non-persisters and persisters, the fecal abundance of *Bifidobacterium* changed by ∆ (−1) – (+3) log_10_ colony-forming units g^−1^ feces after lactose consumption (31), indicating that *Bifidobacterium* are responders to dietary lactose intake, but that the change in abundance differed between individuals (31). Similarly, bifidobacteria were consistently increased here and in other *in vitro* studies (32, 33), confirming that members of the *Bifidobacterium* populations of adults are competitive utilizers of lactose. In addition, in 2 out of the 10 fecal microbiota samples, *Enterobacteriaceae* increased in abundance in the presence of lactose, and active β-galactosidases were detected, in agreement with observations that strains of *E. coli, Klebsiella,* and *Citrobacter* can utilize lactose (34).

### Butyrate formation from lactose does not only rely on cross-feeding

Upon the addition of lactose, we observed consistent alterations of SCFA profiles with higher levels of butyrate. Studies investigating the impact of lactose as a sole carbohydrate source on fecal batch fermentations reported significantly higher acetate and no changes in butyrate or propionate formation (32, 33). Here, we provided lactose together with complex polysaccharides present in Macfarlane medium such as starch, inulin, xylan, arabinogalactan, and mucin (14), mimicking carbohydrate availability that would occur in the lower gastrointestinal tract and allowing for extensive cross-feeding activity.

In our study, the donor microbiota of all 10 donors responded with the formation of more butyrate when supplied with lactose, which was different from the observations of responder/non-responder microbiota observed for more complex dietary fibers (35–37). The response in fermentation activity of xylo-oligosaccharides, inulin, or β-glucan depended on the initial fecal microbiota composition and fermentation type, i.e., butyrogenic or propiogenic (35, 36). For polymeric substrates such as inulin, butyrate production depends on a chain of events, i.e., initial degradation by *Bifidobacterium*, formation of fermentation metabolites, and cross-feeding on acetate and lactate by butyrate producers (37). The higher abundance of the lactate utilizer *Veillonella* suggests that also in this study, lactate-based cross-feeding occurred (38). Yet, our community analyses highlighted that certain gut microbes can both hydrolyze and metabolize lactose to butyrate.

*In vitro,* we confirmed that strains of *A. hallii* and *F. duncaniae,* which are major butyrate producers in the gut (39), expressed β-galactosidase and produced butyrate in the presence of lactose as a sole carbohydrate source. The versatile *A. hallii* can concurrently utilize lactate/acetate to form butyrate, also profiting from lactose-based cross-feeding. Together, our data suggest that lactose availability can lead to butyrate formation by selected butyrate producers without the need to cross-feed.

### How do *in vitro* SCFA profiles relate to *in vivo* observations?

One key feature of our data was the consistent increase in butyrate levels when lactose was supplied during fermentation. Few *in vivo* studies monitored fecal SCFA profiles upon consumption of lactose, and an impact on butyrate levels was rarely mentioned. In one study, the fecal levels of SCFA did not differ after 4-week intervention with whole milk compared to concentrations before the treatment (30). In a cohort of lactase non-persisters, there was no difference in levels of fecal SCFA profiles (presented as average with wide standard deviations) compared to before the intervention (6).

Even in lactase persisters, it is likely that a proportion of lactose remains undigested and becomes available to the intestinal microbiota colonizing the lower gastrointestinal tract. Lactose bioaccessibility also depends on the delivery form, e.g., a liquid matrix or a gel like yogurt (40), and the transport of digesta can be faster than hydrolysis rates, as determined *in vitro* (41). A previous study estimated that up to approximately 8% and 80% of an ingested lactose meal might reach the lower ileum/proximal colon in lactase-persistent and nonpersistent hosts, respectively (42). While microbial metabolism of lactose alone might not be sufficient to trigger a major change in the overall fecal fermentation metabolite profiles, even in lactase non-persisters, our data indicate that lactose fermentation by colonic microbes can contribute to the intestinal butyrate pool. Additionally, concurrent absorption events might occur that can affect fecal SCFA profiles (43).

### *S. thermophilus* had low invasive capacity even when supplied together with lactose

Food-derived microbes can, at least temporarily, constitute a proportion of the intestinal microbial community. Our findings indicate that *S. thermophilus* LMG 18311 was not able to compete with the fecal microbiota even though it was added to constitute about 5% of the population at the beginning of fermentation. Based on an estimation that about 10^10^–10^11^ bacteria cells g^−1^ colonize the colon (44), a starter culture with 10^10^–10^11^ cells, for example, in a portion of yogurt (17), could constitute (at least temporarily) a major proportion of the intestinal microbiota. Data about the survival of *S. thermophilus* during gastrointestinal passage are not without controversy due to the presence of taxonomically closely related streptococci from the oral cavity, e.g., *Streptococcus salivarius*/*Streptococcus vestibularis* group, and the lack of sensitivity in cultivation-based studies (45). However, higher fecal counts of *S. thermophilus* and/or *Streptococcaceae* are frequently reported after yogurt consumption (17, 45).

Before supplementation, *S. thermophilus* cultures were grown in media supplied with lactose under anaerobic conditions to allow for the expression of β-galactosidases and prepare for addition to the fecal microbiota. When added, *S. thermophilus* encountered a fecal microbiota that expressed β-galactosidases even in the absence of lactose. Additional β-galactosidases of *B. obeum* and *C. perfringens* were identified beside enzymes of *Bifidobacterium* spp., *E. coli,* and *A. hallii* when lactose was present, indicating competition with a diverse population contributing the same function.

In this study, *S. thermophilus* was also supplied together with its substrate lactose. Similar to what was observed by Krumbeck et al. (29), who tested the co-application of *Bifidobacterium adolescentis* and *Bifidobacterium animalis* subsp. *lactis* BB12 with lactose-containing β-galacto-oligosaccharides *in vivo,* no synergistic benefits could be observed likely again due to competition with the fecal microbiota. As the final microbiota profiles and *Streptococcaceae* counts were not different between supplemented and unsupplemented samples, and between samples with added wild type or ∆*LacZ* mutant, our data suggest that there was no or low competitiveness of the food culture and that competitiveness could not be enhanced by the addition of a favorite substrate.

### In the presence of lactose, the formation of H_2_S was lower

While H_2_ and CH_4_ breath tests are used as indicators of lactose intolerance (7, 46), there is little information on any connection between lactose intake and H_2_S formation. H_2_S can be produced by sulfate-reducing bacteria like *Desulfovibrionaceae* or from the fermentation of sulfo amino acids (47). In a previous study with a comparable experimental setup, we observed that the addition of fucose reduced H_2_S levels in fecal microbiota fermentations, similar to what was observed here with the addition of lactose (18). Based on *in silico* approaches (47), cysteine degraders, which include *Enterobacteriaceae,* are common within the human microbiota and more abundant than sulfate-reducing bacteria, suggesting that most H_2_S is derived from amino acid metabolism. Our observations here support previous reports that in the presence of easily fermentable substrates like lactose, microbes reduce the utilization of sulfo amino acids, which leads to lower levels of H_2_S (18, 48).

### Conclusion

This study brings forward new insight into the fecal microbial response to a dairy model system consisting of a food-specific disaccharide that was co-supplied with a lactose-utilizing culture. Lactose utilization did not solely rely on cross-feeding, but also on direct metabolism, providing a possibility to selectively enhance the activity of lactose-using butyrate producers. Lactose addition led to major shifts in fermentation systems, affecting the levels of final metabolites (increase in butyrate) and gases (reduction in H_2_S), both effects contribute beneficially to intestinal microbe-host interactions. These results might be highly relevant to the proportion of the population with the lactase non-persistent genotype. We show that dairy starter cultures did not benefit from possessing β-galactosidase within a complex microbial community and that these starters showed little competitiveness, regardless of the presence of β-galactosidase activity.

## Data Availability

16S rRNA gene amplicon libraries are available at PRJEB77001 at ENA. The mass spectrometry data files and mascot search results were deposited to the ProteomeXchange Consortium via the PRIDE partner repository with the data set identifier PXD054534.

## SUPPLEMENTAL MATERIAL

Supplemental figures and tables spectrum.02193-24-s0001.docx Fig. S1 to S5; Tables S1 to S3.

ASM does not own the copyrights to Supplemental Material that may be linked to, or accessed through, an article. The authors have granted ASM a non-exclusive, world-wide license to publish the Supplemental Material files. Please contact the corresponding author directly for reuse.
