# Supplementary material for: In vitro investigations on the impact of fermented dairy constituents on fecal microbiota composition and fermentation activity
Source: Microbiol Spectr. 2025 Feb 4;13(3):e02193-24. doi: 10.1128/spectrum.02193-24 (PMC11878039; doi:10.1128/spectrum.02193-24)
Supplement: Supplemental figures and tables — Fig. S1 to S5; Tables S1 to S3. [file spectrum.02193-24-s0001.docx]

**Supplementary Data** for

***In vitro* investigations on the impact of fermented dairy constituents on fecal microbiota composition and fermentation activity**

Qing Li^1^*, Angeliki Marietou^1^ Freja Foget Andersen^1^, Jiri Hosek^1^, Carsten Scavenius^2^, Jianbo Zhang^3^, Clarissa Schwab^1#^

^1^Department of Biological and Chemical Engineering, Aarhus University, Denmark

^2^Department of Molecular Biology and Genetics, Aarhus University, Denmark

^3^Swammerdam Institute for Life Sciences, University of Amsterdam, the Netherlands

*current affiliation, Technical University of Denmark

^#^corresponding author: schwab@bce.au.dk

Gustav Wieds Vej 10, 8000 Aarhus, Denmark


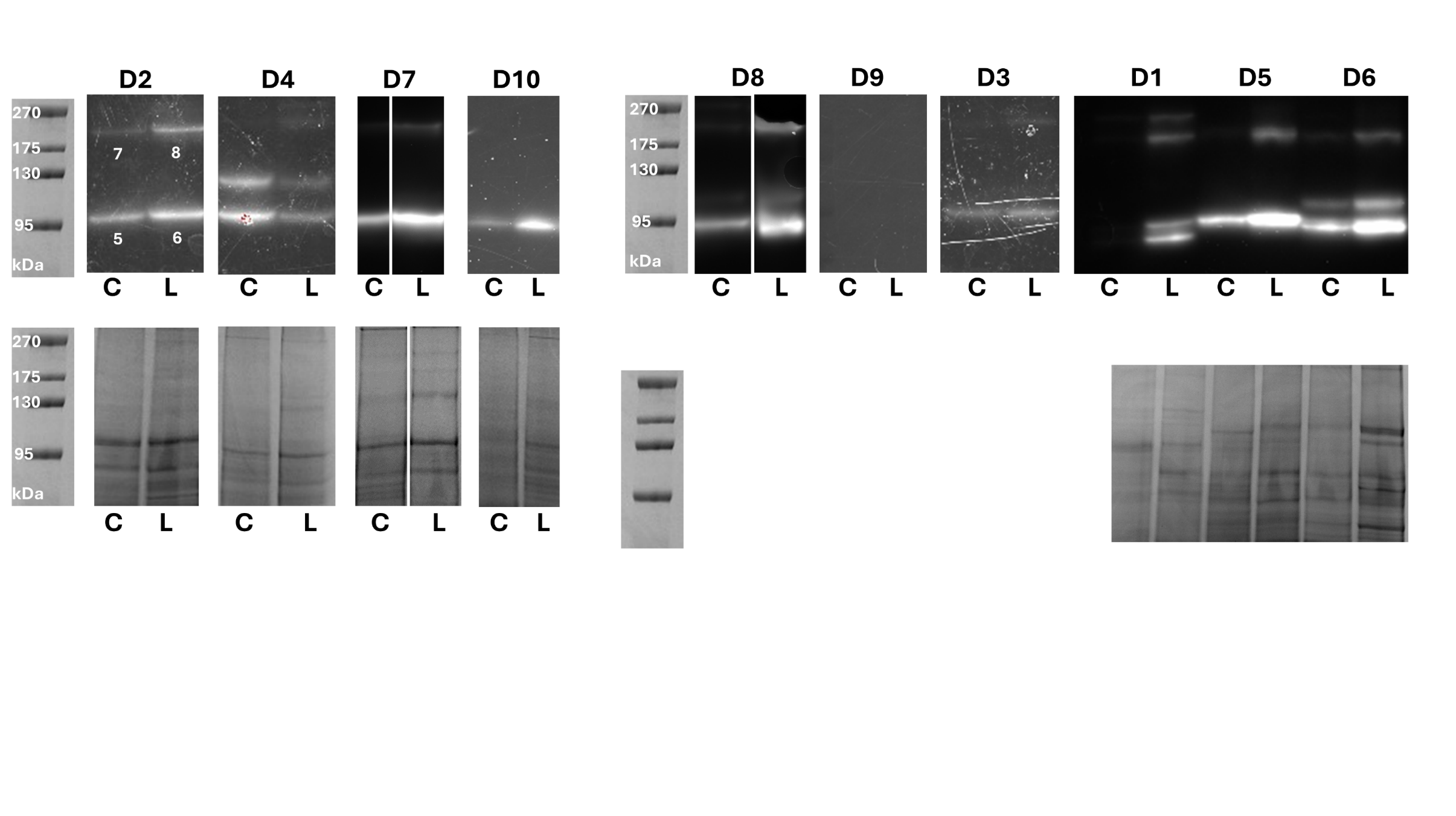


**Suppl. Fig. S1. Activity staining of β-galactosidases.** Proteins extracts were separated using SDS-PAGE and stained with MUG. Shown are activity profiles of different donors with fecal microbiota grown in MF-C (C) and MF-L (L).


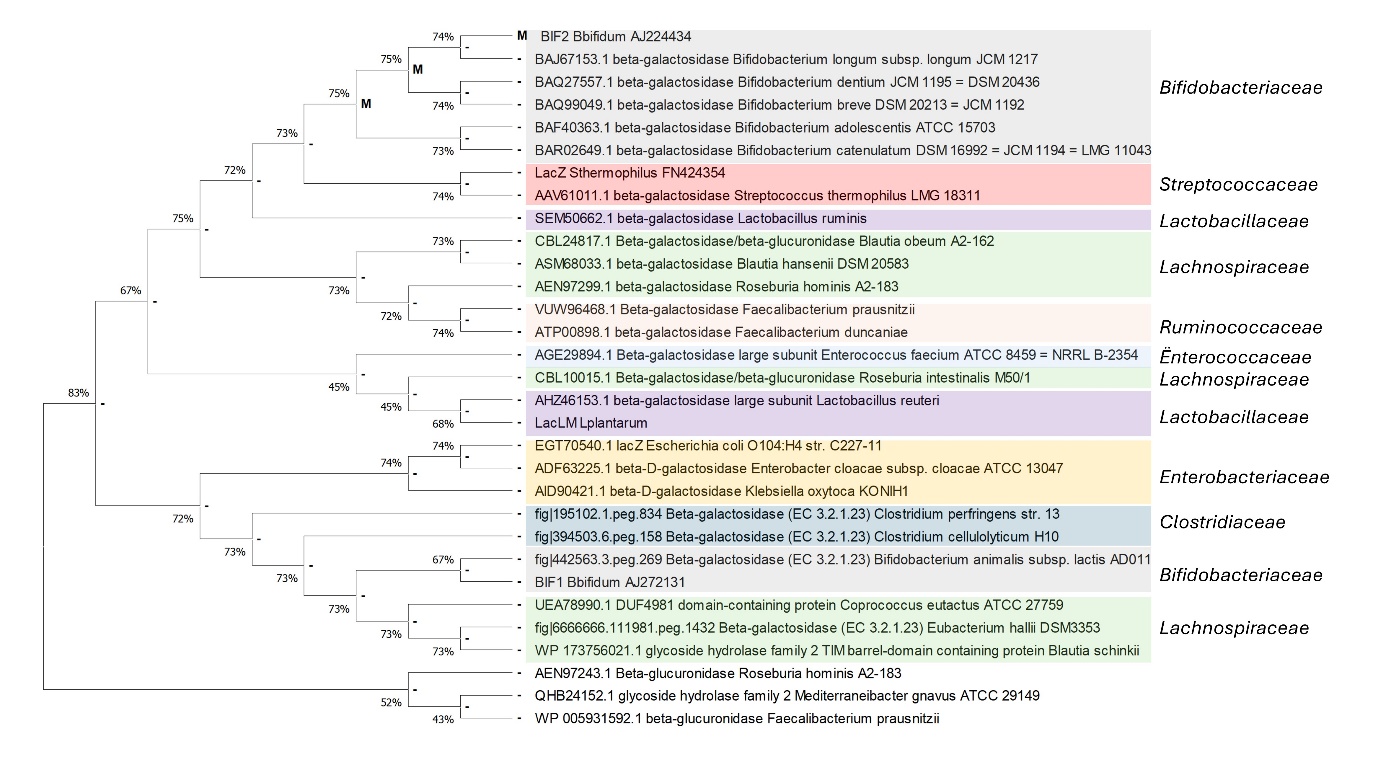


**Suppl. Fig. S2**. **Phylogenetic analysis of β-galactosidase**. Protein sequences of *A. hallii* and *Faecalibacterium* and homologs of gut related species were retrieved from the KEGG database. Sequences from β-glucuronidases (also glycosyl hydrolase family 2, EC 3.2.1.31) were included as outgroup. The amino acid sequences were aligned using BioEdit. The maximum-likelihood phylogenetic tree was inferred using MEGA and 500 bootstraps.


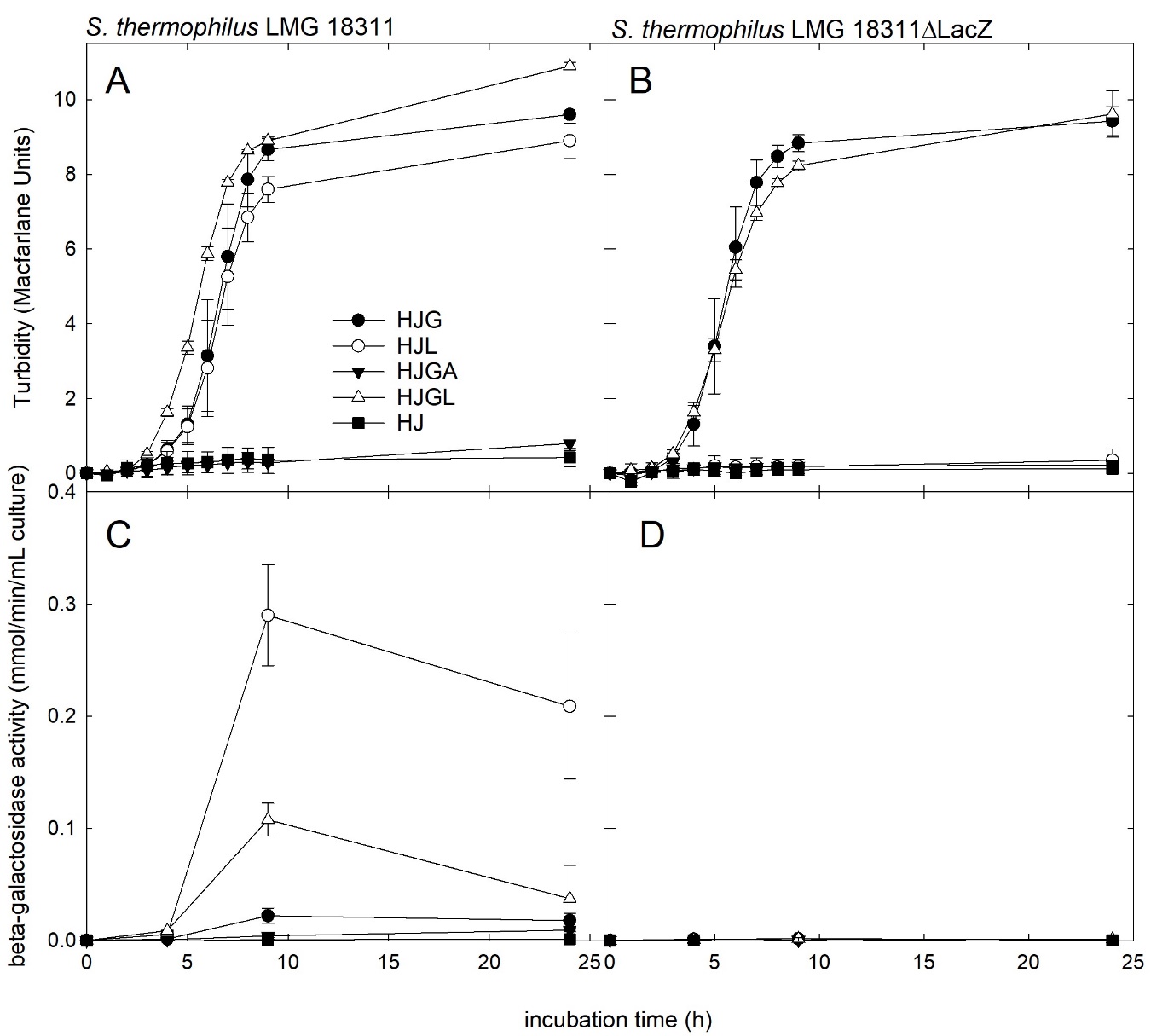
**Suppl. Figure S3. Growth and β-galactosidase activity of *S. thermophilus*.** Strains LMG 18311 (A,C) and LMG 18311∆*LacZ* (B,D) were grown in HJ medium (HJ) supplied with glucose (HJG), lactose (HJL), galactose (HJGA) and lactose and glucose (HJGL) at 42 ºC for 24 h. Turbidity (A,B) was recorded using a Macfarlane densiometer, bet-galactosidase activity (C,D) was measured using oNPG as substrate. Experiments were conducted in three biological independent replicates.


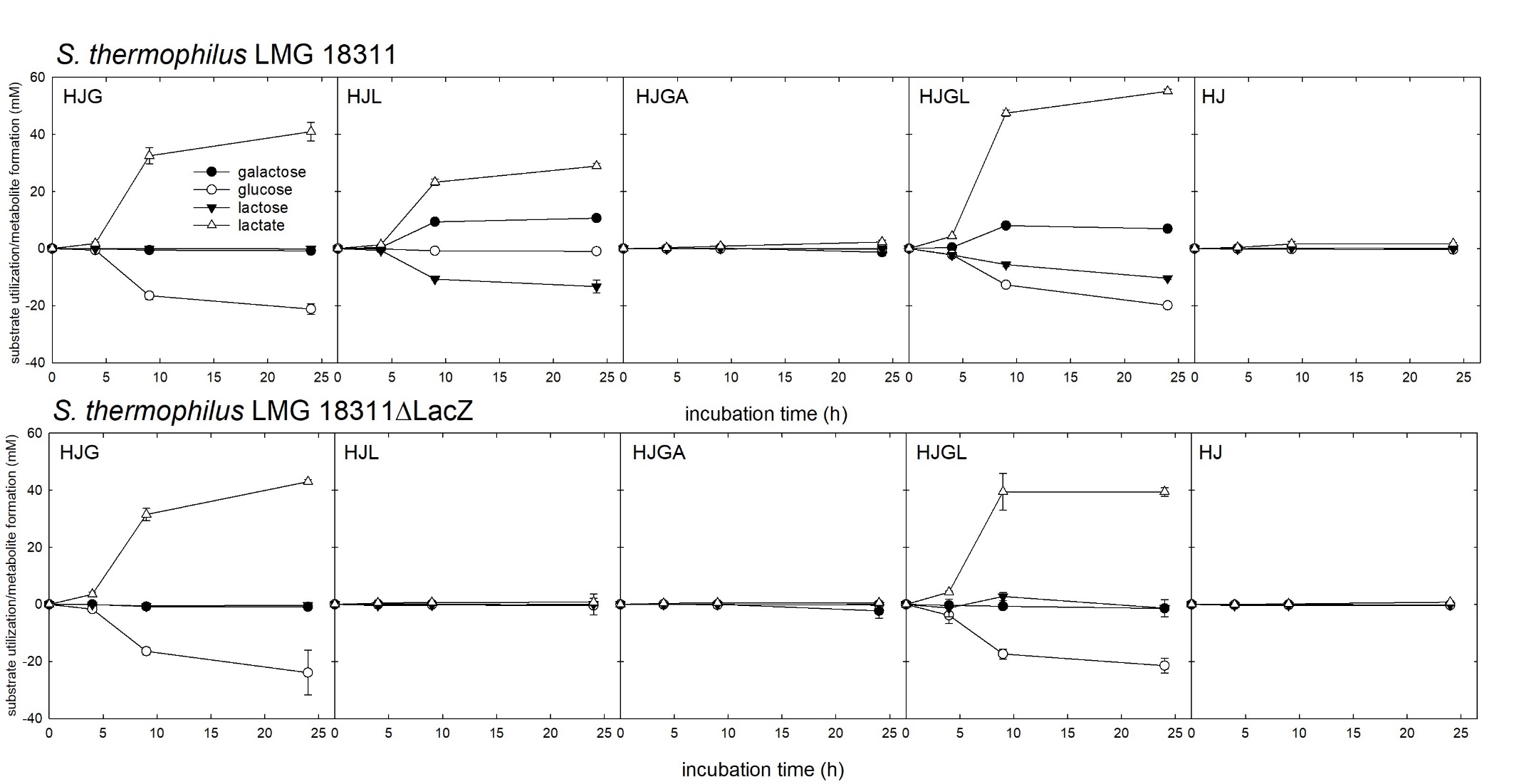
**Suppl. Figure S4. Substrate utilization and metabolite formation of *S. thermophilus* during growth*.*** Strains LMG 18311 (upper panel) and LMG 18311∆*LacZ* (lower panel) were grown in HJ medium (HJ) supplied with glucose (HJG), lactose (HJL), galactose (HJGA) and lactose and glucose (HJGL) at 42 ºC for 24 h. Substrate and lactate concentrations were determined using HPLC-RI. Experiments were conducted in three biological independent replicates.

A


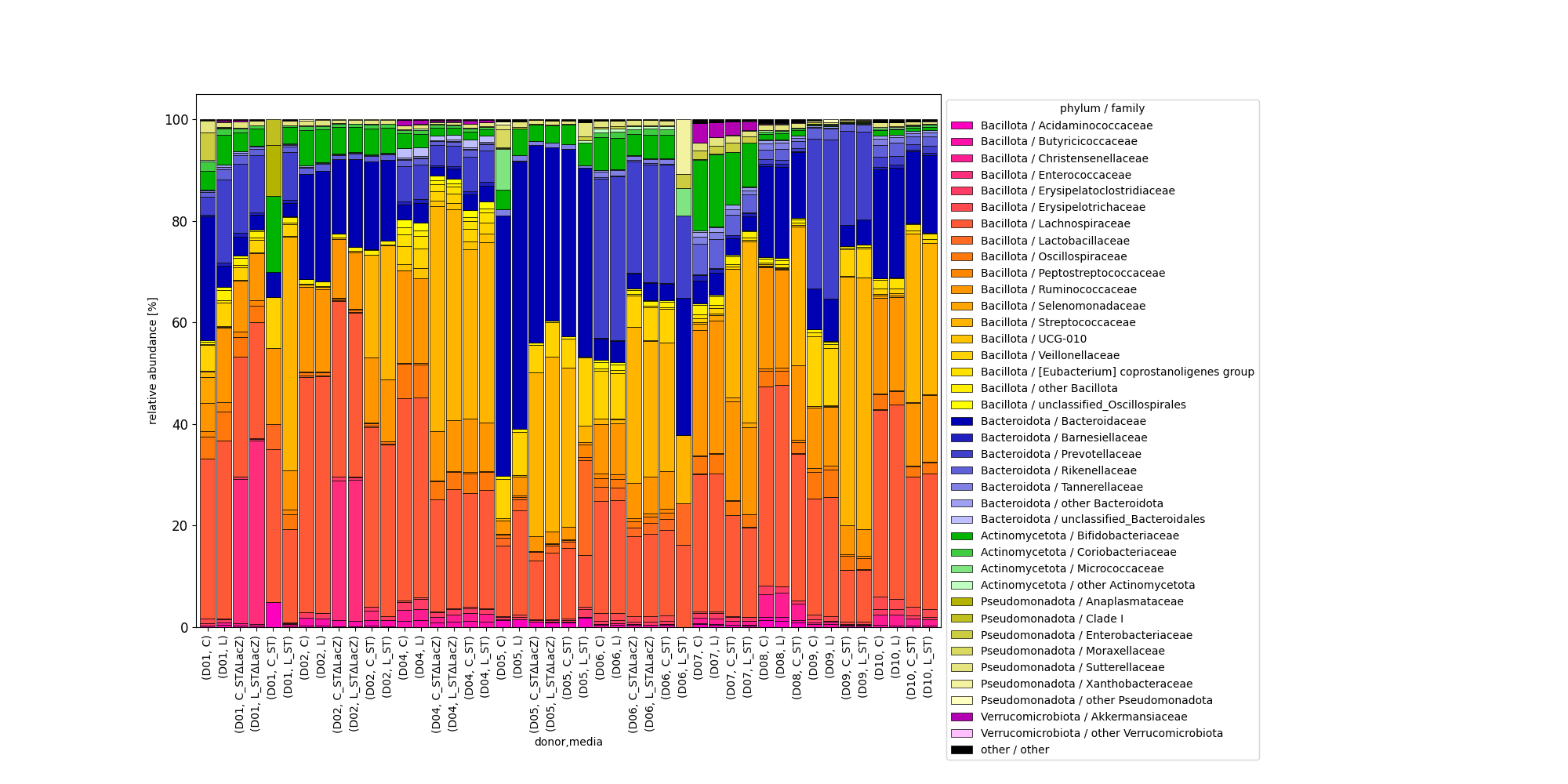


B


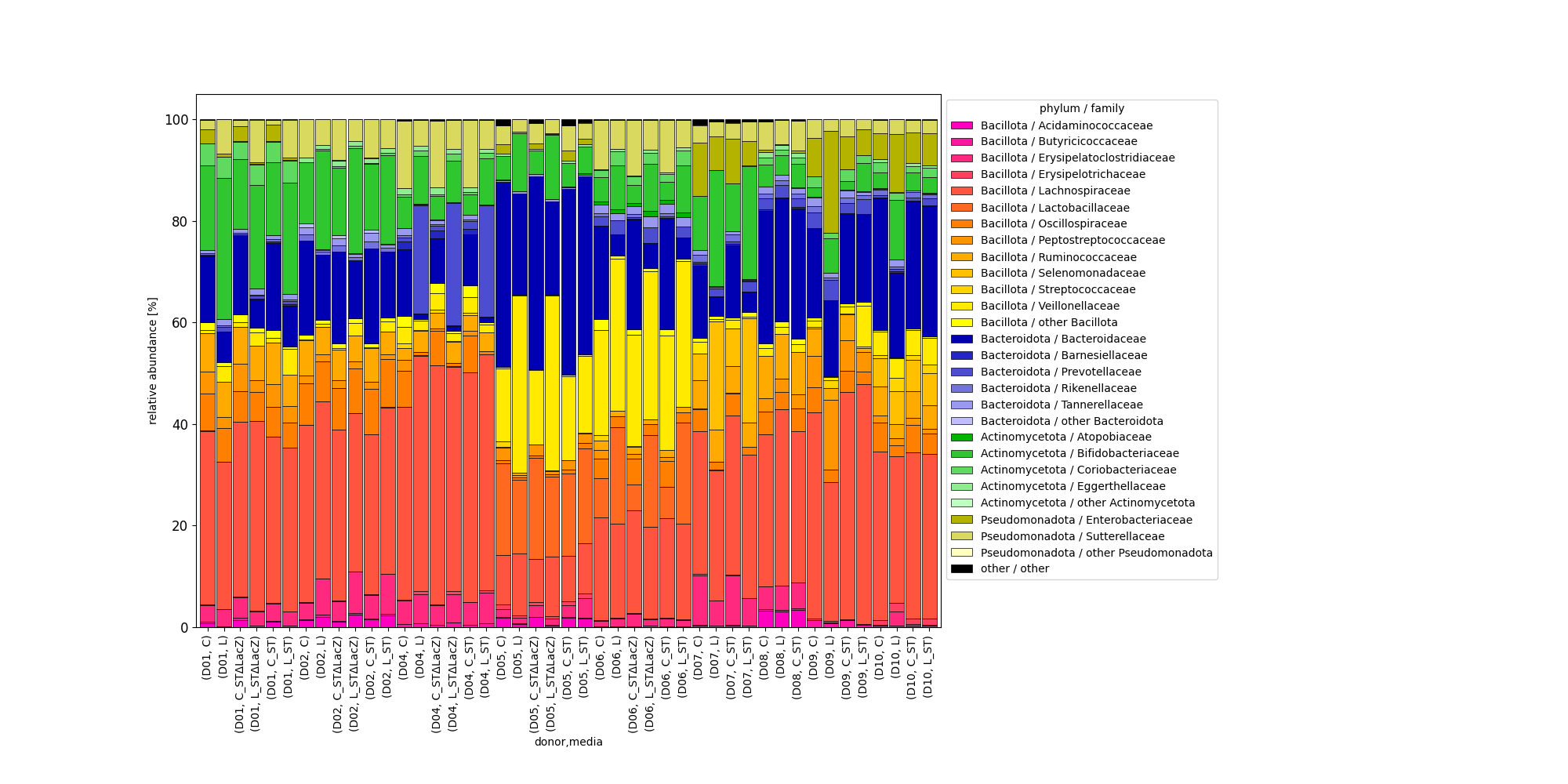


**Suppl. Figure S5. Proportion of bacterial families in fecal fermentation in the presence of *S. thermophilus.*** Microbial community composition was determined using 16S rRNA gene sequencing. Composition was determined at t=0 h (A) and t=48 h (B). At 48 h, both fermentation replicates were sequenced and mean values are shown in the plot.

**Suppl. Table** **S1. Shannon indexes**. Fecal slurries were fermented using Macfarlane medium supplemented with lactose and/or *S. thermophilus* LMG 18311 or LMG 18311∆*LacZ*. 16S rRNA gene sequencing was done using V1-V3 region. Shannon index was calculated using QIIME 2. NT, not tested

|  |  | **Medium composition, treatment** | | | | | |
| --- | --- | --- | --- | --- | --- | --- | --- |
|  |  |  |  | *S. thermophilus* LMG 18311 | | *S. thermophilus* LMG 18311∆LacZ | |
| donor | time (h) | MF-C | MF-L | MF-C | MF-L | MF-C | MF-L |
| D1 | 0 | 6.34 | 6.43 | failed | 4.62 | 5.55 | 5.06 |
|  | 48 | 5.78 | 5.29 | 5.77 | 5.38 | 5.83 | 5.39 |
| D2 | 0 | 5.77 | 5.78 | 5.45 | 5.17 | 5.01 | 5.07 |
|  | 48 | 5.87 | 5.69 | 5.83 | 5.74 | 5.81 | 5.67 |
| D4 | 0 | 6.70 | 6.74 | 5.56 | 5.40 | 4.96 | 5.13 |
|  | 48 | 6.13 | 5.41 | 6.13 | 5.43 | 6.11 | 5.39 |
| D5 | 0 | 5.28 | 5.18 | 4.52 | 5.06 | 4.40 | 4.37 |
|  | 48 | 5.08 | 4.49 | 5.01 | 5.61 | 4.90 | 4.48 |
| D6 | 0 | 6.18 | 6.13 | 5.57 | failed | 5.27 | 5.51 |
|  | 48 | 5.52 | 4.86 | 5.42 | 4.86 | 5.24 | 4.97 |
| D7 | 0 | 6.09 | 6.00 | 5.41 | 4.92 | NT | NT |
|  | 48 | 5.54 | 4.78 | 5.49 | 4.80 | NT | NT |
| D8 | 0 | 6.50 | 6.50 | 5.63 | NT | NT | NT |
|  | 48 | 5.90 | 5.88 | 5.86 | NT | NT | NT |
| D9 | 0 | 5.68 | 5.68 | 3.97 | 3.96 | NT | NT |
|  | 48 | 5.89 | 5.25 | 5.90 | 5.32 | NT | NT |
| D10 | 0 | 6.25 | 6.23 | 5.25 | 5.34 | NT | NT |
|  | 48 | 5.83 | 5.60 | 5.82 | 5.95 | NT | NT |

**Suppl. Table S2**. **Chao indexes**. Fecal slurries were fermented using Macfarlane medium supplemented with lactose and/or *S. thermophilus* LMG 18311 or LMG 18311∆*LacZ*. 16S rRNA gene sequencing was conduced using V1-V3 region. Chao index was calculated using QIIME 2. NT, not tested.

|  |  | **Medium composition/treatment** | | | | | |
| --- | --- | --- | --- | --- | --- | --- | --- |
|  |  |  | | *S. thermophilus* LMG 18311 | | *S. thermophilus* LMG 18311∆*LacZ* | |
| donor | time (h) | MF-C | MF-L | MF-C | MF-L | MF-C | MF-L |
| D1 | 0 | 423 | 342 | failed | 304 | 333 | 304 |
|  | 48 | 272 | 241 | 258 | 231 | 250 | 232 |
| D2 | 0 | 221 | 226 | 234 | 233 | 217 | 228 |
|  | 48 | 203 | 206 | 204 | 209 | 211 | 218 |
| D4 | 0 | 352 | 348 | 283 | 293 | 297 | 316 |
|  | 48 | 255 | 191 | 220 | 228 | 245 | 172 |
| D5 | 0 | 136 | 165 | 135 | 152 | 107 | 143 |
|  | 48 | 138 | 104 | 132 | 202 | 107 | 113 |
| D6 | 0 | 259 | 270 | 258 | failed | 259 | 274 |
|  | 48 | 196 | 179 | 194 | 192 | 189 | 192 |
| D7 | 0 | 333 | 293 | 265 | 246 | NT | NT |
|  | 48 | 241 | 197 | 223 | 191 | NT | NT |
| D8 | 0 | 326 | 320 | 315 | NT | NT | NT |
|  | 48 | 279 | 274 | 275 | NT | NT | NT |
| D9 | 0 | 286 | 261 | 243 | 237 | NT | NT |
|  | 48 | 251 | 246 | 244 | 228 | NT | NT |
| D10 | 0 | 276 | 265 | 257 | 253 | NT | NT |
|  | 48 | 248 | 216 | 251 | 279 | NT | NT |

**Suppl. Table S3**. **Differential abundance of microbial taxa.** Differences in abundance of microbial families and genera were identified using DESeq2, which employs Wald t-test. For multiple test correction the Benjamini-Hochberg false discovery rate was used. An adjust p-value of p_adj_<0.05 was considered significant, p_adj_<0.1 was considered a trend.

| **Taxonomic level** | **Taxonomic group** | **Log2fold change (standard error)** | **p-value** | **p_adj_-value** |
| --- | --- | --- | --- | --- |
| Family | *Desulfovibrionaceae* | -6.79 (1.46) | 3.1*10^-06^ | 0.0002 |
|  | *Bifidobacteriaceae* | 1.15 (0.34) | 0.0008 | 0.0281 |
| Genus | *Bilophila* | -5.32 (1.47) | 0.0003 | 0.0445 |
|  | *Eisenbergiella* | -2.94 (0.86) | 0.0007 | 0.0445 |
|  | *Parasutterella* | -3.17 (0.95) | 0.0008 | 0.0445 |
|  | *Alistipes* | -1.76 (0.59) | 0.0028 | 0.0976 |
|  | UCG-009 | -3.39 (1.15) | 0.0031 | 0.0976 |
